# Editorial: Strategies for Modulating T Cell Responses in Autoimmunity and Infection

**DOI:** 10.3389/fimmu.2020.00208

**Published:** 2020-02-20

**Authors:** María Fernanda Pascutti, Gustavo Javier Martinez, Maria Florencia Quiroga

**Affiliations:** ^1^Department of Immunohematology and Blood Transfusion, Leiden University Medical Center, Leiden, Netherlands; ^2^Center for Cancer Cell Biology, Immunology and Infection, Discipline of Microbiology and Immunology, Rosalind Franklin University of Medicine and Science, North Chicago, IL, United States; ^3^Consejo Nacional de Investigaciones Científicas y Técnicas, Instituto de Investigaciones Biomédicas en Retrovirus y Sida, Universidad de Buenos Aires, Buenos Aires, Argentina

**Keywords:** T cells, animal studies, human studies, immunemodulation, health, disease

T-cell differentiation and effector functions are shaped by the integration of different signals from the environment. The process initiated when the T-cell receptor is engaged is then modulated by activating (costimulatory molecules and cytokines) and inhibitory (checkpoint receptors and cytokines) signals, which have a direct impact on the development or function of effector and/or regulatory T (Treg) cells (1, 2). In pre-clinical human or animal studies, the manipulation of T-cell function has been attempted via numerous different approaches, including (i) manipulation of metabolism (3), (ii) modulation of autophagy, (iii) regulation of epigenetic processes (4), (iv) agonistic or antagonistic therapies engaging cellular receptors (5), (v) the use of cytokines/chemokines to skew immune response profiles, (vi) the expansion/depletion of certain cell type (i.e., depletion of Tregs or expansion of effector T cells specific for a certain pathogen) (6), and (vii) the use of diverse immunomodulating natural or synthetic compounds that could regulate any of the above mentioned aspects of the immune response (7), among others. The goal of the current Research Topic is to highlight some of these approaches and to focus on the diverse ways of modulating T-cell function or frequency in health and disease.

Within this eBook, some authors addressed the potential of diverse strategies, such as manipulating metabolic pathways, autophagy processes, and/or modification of the redox cellular milieu with an impact on T-cell function. Cluxton et al. proposed that the fundamental difference in metabolic requirements of human Tregs and Th17 cells could be used in order to modulate inflammatory responses in human diseases. Similarly, Liang et al. work focused on the effect of sulforaphane (SFN), a compound derived from certain vegetables, as a modulator of the extracellular redox milieu. They observed that SFN induced a pro-oxidative state, consequently inhibiting Th17-related cytokines via targeting of STAT3. They finally stated that SFN could be a novel option for treatment of inflammatory/autoimmune diseases, such as rheumatoid arthritis. Continuing with the importance of metabolism in mediating T-cell function, Nicoli et al. suggested in their review that antigen-presenting cells could be metabolically manipulated through TLR4, TLR9, or STING to induce metabolic and/or autophagy changes in order to subsequently impact on CD8+ T cell responses, while Merkley et al. revisited the influence of autophagy over T-cell responses by modulating several aspects of antigen-presenting cells' function and the impact of this modulation over T-cell signaling, survival, cytokine production, and metabolism.

The impact of post-transcriptional modifications on T-cell responses was explored in a very nice study by Hao et al.. They unraveled, in a rat model of autoimmune hepatitis, the major role of glycosylation on Treg differentiation and the impact of this deficiency on disease development. They demonstrated that deficiency in O-GlcNAc glycosylation affects the Notch function, a signaling pathway previously reported to promote the differentiation and survival of Tregs. As a result, impaired Treg differentiation allowed for increasing CD4+ T cell infiltration in the liver, indicating that modulation of O-GlcNAc glycosylation in this model could be a target for immunotherapy. In a related disease model, Kroger et al. enumerated the various immunotherapies aimed at controlling autoimmunity in type I diabetes, including among other modulation of regulatory T cells and cytokines.

A more traditional approach used by several authors within this eBook consists of underscoring how signaling through various receptors modifies cellular functions in T cells. For instance, Holgado et al. have described for the first time the role of CD32, a low-affinity Fc-gamma receptor, as promoter of human CD4+ T-cell responses that consequently increases cell proliferation and creates a wide pattern of cytokines triggered by polyclonal stimulation, thus enhancing inflammatory responses. Additionally, the work of Jogdand et al. explored the relevance of ICOS signaling in the context of the blood stage of *Plasmodium berghei* ANKA infection. In this setting, they observed that depletion of ICOS+ cells from infected mice induced longer survival and lower parasitemia. Also, they observed that ICOS+ T cells also expressed IFN-γ and the transcription factor Tbet; both positively correlated with ICOS expression, therefore suggesting a positive regulation loop between ICOS expression and T-cell function. Abderrazak et al. observed in their manuscript that VLA-2 integrin signaling protected T cells from methotrexate-induced apoptosis (a drug used for rheumatoid arthritis treatment), and “rescued” cells producing IL-17 and IFN-γ contributing to the perpetuation of inflammation and the development of methotrexate resistance seen in rheumatoid arthritis. Finally, Tosello Boari et al. demonstrated the importance of IL-17 cytokines and their receptor IL-17RA in a model of chronic infection with *Trypanosoma cruzi*. In their work, the authors demonstrated that signaling through IL-17RA is required for the maintenance of effector function in CD8+ T cells in a cell-intrinsic manner. In fact, deficiency in IL-17RA rendered CD8+ T cells more dysfunctional, and blockade of PD-L1, a checkpoint inhibitor currently targeted in many cancer models, partially restored the effector function of these cells. In line with this topic, Panagioti et al. discussed in their review article the various T-cell inducing vaccines used in protection against chronic infections, particularly in viral infections.

Chemokines and/or cytokines themselves could serve as therapeutic targets. In their work, Ushio et al. used a murine model for Sjögren's syndrome as a way to understand the role of CCL22 in this autoimmune disease. They observed that CCL22-producing tissue-resident macrophages may control autoimmune lesions by enhancing the migratory capacity of CD4+ T cells to the affected tissue as well as by inducing IFN-γ production of T cells. Another example of how cytokines and their downstream effects could be used as targets for immunotherapy is addressed in the work of Min et al.. The authors clearly showed the relevance of the Type I IFN/IL-10 axis in the induction of antigen-specific T-cell function and the generation of T-cell memory in a mouse model for scrub typhus, an infectious disease caused by the intracellular bacteria *Orientia tsutsugamushi*. Their work contributes to a better understanding of the mechanisms behind the short longevity of antigen-specific adaptive immunity observed during this human infection (8).

A different approach proposed by some authors consists of expanding effector cells or limiting the function of regulatory cells with the aim to improve immune responses against pathogens. Salido et al. expanded *in vitro* CD8+ T cells from HIV+ subjects on combination antiretroviral therapy (cART). Their ultimate goal was to explore new strategies aimed at modulating CD8+ T lymphocytes to achieve functional cure of HIV infections. They observed that expanded cells were polyfunctional, skewed toward an effector phenotype, and that PD-1 expression was clustered in HIV-specific effector memory CD8+ T cells, which had the highest cytokine-producing capacity. Antiviral activity was also enhanced in these cells, indicating that, despite being dampened in subjects on cART, the HIV-specific CD8+ T-cell response could be selectively stimulated and expanded *in vitro*. On the other hand, the work of Araujo Furlan et al. explored the role of Tregs during *Trypanosoma cruzi* infection and the impact of Treg frequency over pathology. They observed that massive accumulation of effector immune cells resulted in a diminished frequency of Tregs and inversely correlated with the magnitude of the immune response as well as with emergence of acute immunopathology. After transfer of Tregs, CD8+ T-cell function was affected and parasite control in blood and tissues was diminished, highlighting the relevance of Tregs over cytotoxic immunity against the parasite. Another important population of T cells that was reviewed by Shiromizu et al. consists of γδ T cells, whose role during autoimmunity and infection has been analyzed in depth in their work. They have also revised concepts about the mechanisms that modulate γδ T-cell function, giving the framework to consider the modulation of these cells as a tool to control pathological immune responses.

Collectively, the wide spectrum of original papers and reviews presented in this eBook have provided a comprehensive overview of potential strategies aiming to convert a pathological immune response into a protective one through the control or reprogramming of specific T-cell subsets. The insights outlined here will hopefully help shape future therapeutics for the treatment of infectious and autoimmune diseases.

## Author Contributions

MQ wrote the first draft of the present Editorial article. MP and GM revised it and provided their valuable and precious comments, remarks, and suggestions. The final Editorial's draft and the current Editorial's text were entirely agreed and approved by all three Guest co-Editors of the present Research Topic, and it details the Strategies for Modulating T cell Responses in Autoimmunity and Infection.

### Conflict of Interest

The authors declare that the research was conducted in the absence of any commercial or financial relationships that could be construed as a potential conflict of interest.
